# Microvascular Sex- and Age- Dependent Phosphodiesterase Expression

**DOI:** 10.3389/fragi.2021.719698

**Published:** 2021-07-27

**Authors:** Jianjie Wang, Murtaza M. Kazmi, Virginia H. Huxley

**Affiliations:** ^1^ Department of Biomedical Sciences, Missouri State University, Springfield, MO, United States; ^2^ Department of Medicine, The Aga Khan University, Karachi, Pakistan; ^3^ Department of Medical Pharmacology and Physiology, National Center for Gender Physiology, Dalton Cardiovascular Research Center, Columbia, MO, United States

**Keywords:** phosphodiesterase, arterioles, venules, sex, age, adult, juvenile, skeletal muscle

## Abstract

**Objective:** The cyclic nucleotide second messengers, cAMP and cGMP, are pivotal regulators of vascular functions; their cellular levels are tightly controlled by the cyclic nucleotide hydrolases, phosphodiesterases (PDE). Biologic sex and age are recognized as independent factors impacting the mechanisms mediating both vascular health and dysfunction. This study focused on microvessels isolated from male and female rats before (juvenile) and after (adult) sexual maturity under resting conditions. We tested the hypothesis that sexual dimorphism in microvascular PDE expression would be absent in juvenile rats, but would manifest in adult rats.

**Methods:** Abdominal skeletal muscle arterioles and venules were isolated from age-matched juvenile and adult male and female rats under resting conditions. Transcripts of five PDE families (1–5) associated with coronary and vascular function with a total of ten genes were measured using TaqMan real-time RT-PCR and protein expression of microvessel PDE4 was assessed using immunoblotting and immunofluorescence.

**Results:** Overall expression levels of PDE5A were highest while PDE3 levels were lowest among the five PDE families (*p* < 0.05) regardless of age or sex. Contrary to our hypothesis, in juveniles, sexual dimorphism in PDE expression was observed in three genes: arterioles (PDE1A, female > male) and venules (PDE1B and 3A, male > female). In adults, gene expression levels in males were higher than females for five genes in arterioles (PDE1C, 3A, 3B, 4B, 5A) and three genes (PDE3A, 3B, and 5A) in venules. Furthermore, age-related differences were observed in PDE1-5 (in males, adult > juvenile for most genes in arterioles; in females, adult > juvenile for arteriolar PDE3A; juvenile gene expression > adult for two genes in arterioles and three genes in venules). Immunoblotting and immunofluorescence analysis revealed protein expression of microvessel PDE4.

**Conclusion:** This study revealed sexual dimorphism in both juvenile and adult rats, which is inconsistent with our hypothesis. The sex- and age-dependent differences in PDE expression implicate different modulations of cAMP and cGMP pathways for microvessels in health. The implication of these sex- and age-dependent differences, as well as the duration and microdomain of PDE1-5 activities in skeletal muscle microvessels, in both health and disease, require further investigation.

## Introduction

Acceptance of sex as a biological variable has led to initiation of understanding of a wide range of disparities between women and men in health and especially in diseases from epidemiology, pathophysiology, clinical manifestations, disease progression, and response to treatment (Mauvais-Jarvis et al., 2020). Cardiovascular disease remains the leading cause of death in the United State where mortality is higher for women than men (Gulati et al., 2012). As a consequence of studies focusing on the reproductive hormones, *via* both genomic and non-genomic mechanisms, sex steroids have been shown to mediate some but not all of the sex-differences noted in adult patients and several animal models with cardiovascular disease. Of interest, a role for biologic sex with respect to cardiovascular function has been found at a stage of life, before the production of reproductive hormones, when sex hormone levels are comparable (Huxley, 2007; Huxley and Wang, 2010; Intapad et al., 2014; Winham et al., 2015). Among the observations in pre-adolescent humans, are the worse cardiovascular outcomes and earlier death for girls who developed type 1 diabetes at <10 years of age relative to comparable boys (Rawshani et al., 2018). Sex, independent of age, has also been identified as a social determinant of cardiovascular risk (O’Neil et al., 2018). Finally, in the clinical realm, it has been recognized that diagnosis and therapeutic treatment regimens tailored specific to sex- and age- for cardiovascular diseases need to be established if effective personalized medicine is to be achieved.

To date, limited knowledge exists for understanding the mechanisms regulating cardiovascular responses in males and females as children, adults, or during old age. Our previous *in vivo* and *ex-vivo* studies on vascular barrier function demonstrated differences between age-, origin, and species-matched microvessels of adult as well as juvenile animals (Huxley et al., 2005; Wang et al., 2006; Huxley et al., 2007; Wang and Huxley, 2007; Huxley et al., 2018). Little is known of the regulatory mechanisms of sex-differences in the heart and vascular system. This paucity of knowledge contrasts with studies on the brain where sex has been shown to influence numerous key cerebral structures, neurotransmitters, and cell signaling molecules across the lifespan from embryo to old adults (Xu and Disteche, 2006; McEwen and Milner, 2017; Ratnu et al., 2017). This present study was designed to identify the influence of sex as a biological variable in genetic expression of phosphodiesterase, a key cellular enzyme that influences directly the levels of the second messengers, cAMP and cGMP, in intact arterioles and venules of skeletal muscle in juvenile and adult rats.

Phosphodiesterases (PDE), key intracellular enzymes hydrolyzing cyclic nucleotides (cAMP and cGMP), are found in most tissues in the body. There are a total of 11 PDE families encoded by 21 genes; as a consequence of splice variants, these families contain 60^+^ isoenzymes of PDE (Omori and Kotera, 2007). Among the 11 PDE families, PDE1-5 play pivotal roles in the regulation of vascular function, as evidenced by selective PDE1-5 inhibitors modulating vascular tone, exchange, and remodeling through cAMP and cGMP-mediated signaling processes in endothelial (Netherton et al., 2007) and smooth muscle cells (Houslay et al., 2007). Thus, expression of 10 isoenzymes of PDE1-5 families was investigated in intact arterioles and venules herein.

Following their discovery in the 1970s, PDEs became therapeutic targets only a decade later. For instance, theophylline, naturally found in cocoa beans and teas, induces vascular smooth muscle relaxation through elevation of cAMP level by inhibiting PDE activity. Most recently, highly selective PDE3, PDE4, and PDE5 inhibitors have been developed as novel treatments for pulmonary hypertension, inflammation, and ischemic diseases (Francis et al., 2011; Page and Spina, 2011). However, fundamental characteristics of PDE in intact vessels, especially those mediating microvascular resistance, blood flow distribution and tissue exchange, remain poorly investigated. Without such basic knowledge, designing new therapeutic drugs targeting PDE in the context of treatment efficacy with minimal adverse effects will be limited.

The second messengers, cAMP and cGMP, play key roles controlling multiple vascular functions. In arterioles, whose state of contraction sets vascular tone and, subsequently, tissue perfusion (Scallan et al., 2010). cAMP regulates vasodilation induced by G-protein coupled receptor activation and changes in shear stress (Lezoualc’h et al., 2016). For the atrial natriuretic peptides (ANP, BNP, CNP)- and nitric oxide (NO)-dependent dilatators it is the levels of cGMP that mediate changes in arteriolar tone (Kuhn, 2016). Much less is known about the role of the PDE in venular microvessel function. The venules collect blood that has coursed through the capillary networks fed by the arterioles and constitute the major vascular volume reservoir of the body. These low-pressure vessels, while less studied than their arteriolar counterparts play a major role not only in the regulation of vascular volume distribution but also in the regulation of the traffic of larger solutes such as proteins and hormones between the vascular and extravascular spaces. Further, it is in the venular vessels where most white blood cells interact (Scallan et al., 2010). Our studies have demonstrated functional role for the PDEs in venular function with respect to microvascular permeability responses that differs from that observed in the arterioles. Hence, we included venules in the present work.

We tested the hypothesis that sex differences in microvascular PDE expression would be absent in juveniles, but displayed in adults. We isolated intact arterioles and venules from abdominal wall skeletal muscles of four groups of rats: adult males, adult females, juvenile males and juvenile females. The PDE1-5 mRNA in intact arterioles and venules was measured quantitatively using the TaqMan method of real-time RT-PCR.

## Materials and Methods

### Experimental Animals and Tissue

All animal care and experiments were conducted in accordance with the National Institutes of Health’s “Guide for the Care and Human Use of Laboratory Animals” under the supervision of the Office of Laboratory Medicine at the University of Missouri-Columbia. The Institutional Animal Care and Use Committee (IACUC) approved all experimental protocols. All Sprague-Dawley Rats (Hilltop Lab Animals, Scottsdale, PA) were divided into four groups by age and sex: adult (age > 3 months) males and females, and juvenile males (age < 60 ± 2 days) and juvenile females (age < 40 ± 2 days). Abdominal muscle was chosen for these studies to facilitate age and sex comparisons while maintaining a link to the extensive literature on skeletal microvascular flow control and oxygen transport conducted in the cremaster muscle. The cremaster muscle, which raises and lowers the testes and is exclusive to males, is derived developmentally from the abdominal skeletal muscle.

### Isolation of Rat Skeletal Muscle Microvessels

All dissecting tools were decontaminated with RNaseZap (Invitrogen, Carlsbad, CA) prior to use. On the day of microvessel isolation, the animals were anesthetized with an intraperitoneal injection of 130 mg kg^−1^ thiobutabarbital (Inactin™, Sigma, St. Louis, MO). Following the removal of fur and an incision made along the middle line of the anterior abdomen skin, the abdominal wall muscle was excised carefully using sterile procedures. The excised piece of abdominal wall (40–50 by 30–40 mm) was placed in RNAlater RNA stabilization solution (Invitrogen, Carlsbad, CA) and microvessels were dissected from the internal surface of the abdominal muscle (*transversus abdominis muscle*) with the aid of a dissecting microscopy (Zeiss, Thornwood, NY). The microvasculature is surrounded by connective tissue that separates the vessels from the adjoining muscle cells so when isolating the vessels from the myocytes opening fine forceps in the connective tissue interspace allows for separation of the vessels from the myocytes. To preserve the integrity of RNA for transcription assay, the tissue was immersed in RNAlater. The microvessels were easily separated from surrounding connective tissue after RNAlater permeabilized tissue, which helped to further remove connective tissue in the samples.

In skeletal muscle, arterioles and venules run parallel to one another. There are several distinctive characteristics between two types of vessels, including general lack of vessel tone and more elliptical profile for venules relative to arterioles. Two major properties, which we used to differentiate them in excised skeletal muscles, include diameter (venule > arteriole) and thickness of vascular wall (arteriole > venule). An arteriolar plexus contained arterioles < 100 µm in internal diameter (ID) that branched from larger feed arteries of the cranial or caudal epigastric artery. Therefore, based on the rat’s sex and reproductive maturity, the isolated arterioles were divided into four groups. The same approach was used for the isolation and characterization of the venules as the venular tree typically runs in parallel to the arterioles. The isolated arterioles and venules were stored in nuclease-free tubes containing the RNAlater solution at 4°C for next day RNA extraction.

### Quantitative Real-Time RT-PCR

Total RNA was extracted from isolated microvessels with RNeasy fibrous tissue kit, including proteinase k treatment (10 µL/reaction), according to the manufacturer’s instruction (QIAGEN, Valencia, CA). The quantity and quality of total RNA were assessed using NanoDrop™, 2000c (Thermo Scientific, Wilmington, DE). To synthesize first strand cDNA, 2 µg of the clean and DNase-treated total RNA that had > 1.8 of an optical density (OD)_260/280_ was reversely transcribed to cDNA using Superscript III, reverse transcriptase, in a total volume of 30 µL following the manufacture’s instruction (Invitrogen, Carlsbad, CA). Amplification of cDNA was performed on a real-time quantitative PCR BioRad IQ5 (Bio-Rad, Hercules, CA). TaqMan® Gene Expression Assay (Applied Biosystems™, CA) was used for PDE real time PCR analysis: PDE1A, Rn00578422_m1; PDE1B, Rn00575591_m1; PDE1C, Rn00579334_m1; PDE2A, Rn00579346_m1; PDE3A, Rn00569192_m1; PDE3B, Rn00568191_m1; PDE4A, Rn00565354_m1; PDE4B, Rn00566785_A1; PDE4D, Rn00566798_m1; and PDE5A, Rn00592185_m1. ß-actin (Rn00667869_m1) served as the internal control because it has been accepted that ß-actin mRNA expression remains relatively constant under a variety of conditions. Rat brain was used as a positive control for PDE expression when we optimized assay conditions (data not shown). To exclude external contamination in the reaction components, no template controls (cDNA template replaced by DEPC-treated H_2_O) served as a negative control. Amplification of cDNA was performed on a real-time quantitative PCR BioRad IQ5 (Bio-Rad, Hercules, CA) for 40 cycles with denaturation at 95°C for 15 s, annealing at 60°C for 1 min, and elongation at 72°C for 1 min following manufacture’s instruction.

### Cell Lysate Preparation and Immunoblotting Assay

Protein extraction from isolated abdominal skeletal muscle arterioles and venules was performed on ice as described previously (Wang and Huxley, 2006; Wang et al., 2010). Briefly, each sample containing two to three arterioles (ID < 100 μm, 1–2 mm in length) or venules (ID < 250 μm, 1 mm in length) was incubated with 20 µL of Laemmli buffer containing 62.5 mM Tris-HCL (pH = 6.8), 6 M urea, 2% SDS (vol/vol), 160 mM dithiothreitol (DTT) and Halt® protease inhibitor cocktail (1:100 dilution, Pierce, Rockford, IL). The sample was boiled for 2 min and sonicated for 10 s twice. The same procedure was repeated 4 times. The lysates were obtained from the supernatant following 15 min centrifugation at 14,000 rpm at 4°C. The proteins were separated by electrophoresis on 4–12% (w/v) Nupage gel (Invitrogen, Carlsbad, CA) and then transferred to PVDF membrane in accordance with the manufacturer’s instructions. The PVDF membrane was incubated overnight at 4°C with primary polyclonal antibody against the pan-PDE4 family (PD4-101AP, 1:500 dilution in 5% BSA-blocking buffer, FabGennix International Inc., Frisco, TX). The PVDF membrane was then incubated with secondary goat anti-rabbit antibody (1: 1,500 dilution) for 1 h at room temperature. The immunoblotting was detected using chemiluminescence substrate SuperSignal West Dura (Pierce, Rockford, IL) and exposed to x-ray film. The pan-PDE4 antibodies have been successfully used for immunoblotting in rat tissue (Giorgi et al., 2004; Mongillo et al., 2006; Fujita et al., 2007).

### Immunofluorescence Assay

The cellular distribution of PDE4A in microvessels *in situ* was determined using dual-color immunofluorescence and confocal laser-scanning microscopy as described previously (Wang and Huxley, 2006; Wang et al., 2010). In brief, cryostat sections (7 µm in thickness) of abdominal skeletal muscle were fixed in methanol-acetone mixed solution (1:1, v/v, −20°C for 10 min). Sections were washed with 0.1 M phosphate buffer saline (PBS, pH 7.4) and blocked with 5% (v/v) normal goat serum (Jackson ImmunoResearch Laboratories, West Grove, PA) diluted in PBS for 1 h. Sections were then incubated with dual primary antibodies, anti-PDE4A polyclonal antibodies (1:250 dilution in 5% w/v BSA-blocking buffer, FabGennix International Inc., Frisco, TX) and anti-CD31 (PECAM) monoclonal antibody (1:50 dilution; Serotec Immunological Excellence, Raleigh, NC) at room temperature for 1 h, followed by three washes with PBS. Secondary antibodies were Alexa Fluor™ 488 labeled goat anti-rabbit IgG (10 μg ml^−1^; Molecular Probes, Eugene, OR) for PDE4A antibody and Alexa Fluor™ 568 labeled goat anti-mouse IgG (6 μg ml^−1^; Molecular Probes, Eugene, OR) for CD31 antibody. Secondary antibodies were applied for 1 h, sections were washed with PBS (× 3), mounted with anti-fading medium (MOWIOL® 488, Calbiochem, San Diego, CA) and viewed with confocal (Radiance 2000™ Confocal Microscopy System, Zeiss, Thornwood, NY) laser (Krypton-Argon)-scanning microscopy in the Cytology and Molecular Core of University of Missouri-Columbia. Immunofluorescence negative controls for secondary antibodies at the concentration used in this study were tested previously with omission of primary antibodies (Wang and Huxley, 2006).

### Statistical Analyses

All values are expressed as mean ± SEM and statistical analysis was performed using GraphPad Prism version 5.02 (Graph Pad Software, San Diego, CA). The samples with cycle threshold (Ct) value > 22 for ß-actin, housekeeping gene, were excluded because the loading level of first strand cDNA was considered to be lower. The level of each PDE mRNA expression was normalized to ß-actin mRNA levels obtained from the same sample in the same PCR reaction. The fold-change relative to ß-actin was calculated by 2^−ΔCt^, where ΔCt = Ct_target_–Ct_ß-actin_. The one-way ANOVA followed by Bonferroni post-hoc test was performed to test differences among five families of PDE 1-5 mRNA expression within each group of rats. To determine sex-specific or reproductive maturity-dependent differences, the comparative quantification of gene expression analysis (2^−ΔΔCt^ method) was employed. The fold-change between males and females was assessed using 2^−ΔΔCt^, where ΔΔCt = ΔCt (male)–ΔCt (female) if expression level in males was greater compared with females and vice versa. The null hypothesis is ΔΔCt = 0 or ΔCt (male) = ΔCt (female). The standard deviation of ΔΔCt was calculated in accordance with the instruction from Applied Biosystems ™ (Applied Biosystem, 2008). One-sample *t*-test by comparing the mean of each fold-difference with hypothetic value of one was used to analyze sex-specific differences. Likewise, to assess fold-change between adult and juvenile, the comparative quantification of gene expression analysis (2^−ΔΔCt^ method) was used, where ΔΔCt was calculated by using the relatively high expression ΔCt subtracting low expression ΔCt for each gene. Statistical significance was defined as *p <* 0.05.

## Results

### Transcript Levels of PDE1-5 Ten Isoenzymes Normalized to β-actin

**FIGURE 1:**
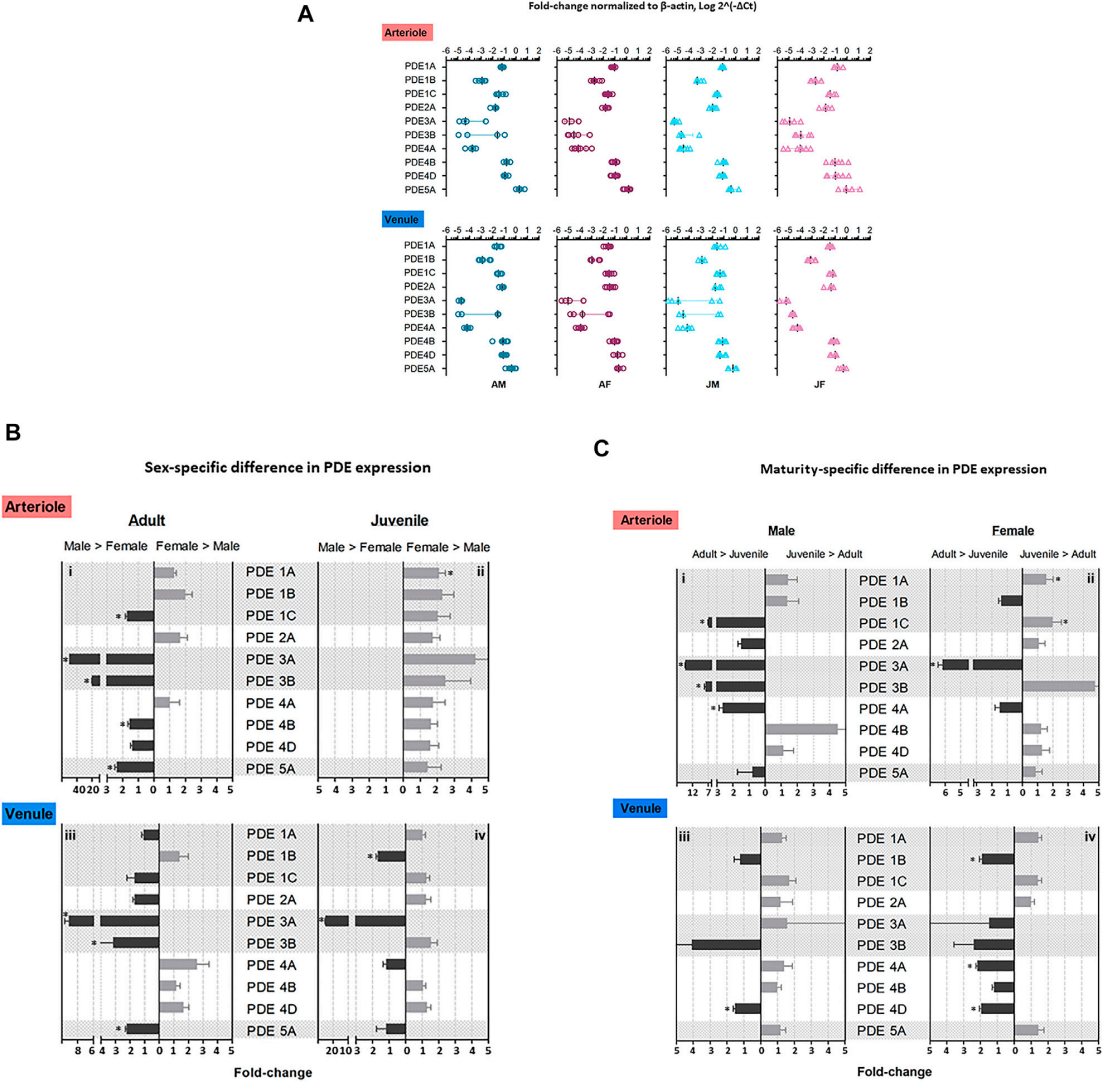
Expression level of PDE1-5 transcripts in adult and juvenile skeletal muscle arterioles and venules of male and female rats. Arterioles and venules were isolated from abdominal skeletal muscles of four group rats: adult males (AM), adult females (AF), juvenile males (JM), and juvenile females (JF). Microvessel PDE 1-5 mRNA expression was measured by using TaqMan real-time RT-PCR assay. **(A)** The fold-change relative to β-actin for PDE gene expression was calculated as 2^−ΔCt^, where ΔCt = Ct_target gene_–Ct_β-actin_. PDE5A mRNA levels were the highest among ten genes in arterioles and venules in each group (*n* = 4–7, ^*#*^ *p* < 0.05). The order of expression levels in PDE1 family: 1A≈1C > 1B; likely for PDE4 family, 4B ≈ 4D > 4A. Expression levels of PDE3 family were relatively low vs. other families, in which there was no significant difference between PDE3A and PDE3B although there was a trend of low expression for PDE3A. These expression patterns were similar for both arterioles and venules in four group rats. **(B)** Sex-specific difference in PDE 1-5 mRNA levels. The relative fold-change between males and females were calculated by 2^−ΔΔCt^, where ΔΔCt = ΔCt_(high expression)_–ΔCt_(low expression)_. In arterioles, levels of PDE1C, 3A, 3B, 4B, 5A were greater in adult males compared with adult females **(i)**; PDE1A level was greater in juvenile females compared with juvenile males **(ii)**. In venules, expression levels of PDE3A, 3B, and 5A were higher in adult males compared with adult females **(iii)**; PDE1B and 3A were higher in juvenile males compared with juvenile females **(iv)**. **(C)** Reproductive maturity-specific difference in PDE 1-5 mRNA level. Using the similar analysis to sex-specific difference, the fold-change between adult and juvenile with the same sex was expressed as 2^−ΔΔCt^. In arterioles, expression levels of PDE1C, 3A, 3B, 4A were greater for adult males compared with juvenile males **(i)**; PDE1A and 1C expression for juvenile females exceeded the counterparts for adult females and only PDE3A level was greater for adult females relative to juvenile females **(ii)**. In venules, PDE4D was greater in adult males relative to juvenile males **(iii)** whereas adult females expressed higher levels of PDE1B, 4A, and 4D relative to juvenile females **(iv)**. The data are means ± SEM of fold-changes. Experimental replication was 4–7 (*n* = 4–7) and * indicates *p* < 0.05.

TaqMan real-time quantitative PCR assay revealed relative expression levels of 10 isoenzymes (PDE1-5). The expression levels of PDE normalized to β-actin were compared among 10 isoenzymes within each group of rats. PDE5A expression was greater relative to the other nine genes for both arterioles and venules, respectively, in each group (*n* = 4–7, *p* < 0.05). PDE3A, PDE3B, and PDE4A expression levels were relatively low among 10 PDE isoenzymes (Figure 1A).

Within each PDE family the expression levels were compared. For PDE1 the levels of PDE1A and PDE1C were higher than that of PDE1B (*p* < 0.05). The expressed levels of PDE4B and PDE4D were much higher than PDE4A (*p* < 0.05). Both PDE3A and PDE3B were expressed in relatively low level and no difference between them was observed (Figure 1A). The expression patterns were similar in both arterioles and venules in the four groups of rats.

### Sex-Specific Differences in PDE1-5 mRNA Expression

Sex-specific differences in the 10 isoenzymes (PDE1-5) was assessed within adults and juveniles separately. The relative mRNA expression level was defined as high level relative to low level. For instance, ΔΔCt = ΔCt_male_—ΔCt_female_ when male’s levels were greater than female’s and vice versa.

In arterioles, genes expressed at greater levels in adult males compared with adult females were PDE1C, PDE3A, PDE3B, PDE4B, PDE5A (male > female in fold-change: PDE1C, 1.72 ± 0.11; PDE 3A, 50.00 ± 0.04; PDE3B, 20 ± 0.11; PDE4B, 1.56 ± 0.10; PDE5A, 2.38 ± 0.12; *p* < 0.05, *n* = 4–7, Figures 1Bi). A trend that did not reach statistical significance for higher expression in adult females relative to adult males was observed in genes PDE1A, PDE1B, and PDE2A. For juveniles, only a single isoenzyme, PDE1A in females exceeded that in males (juvenile female > juvenile male, 2.11 ± 0.37-fold, *n* = 5–6, *p* < 0.05) although there were trends that the levels of all the other isoenzymes to be higher in juvenile females than juvenile males (Figures 1Bii).

In venules, PDE3A, PDE3B, and PDE5A expression were higher in adult males compared with adult females (male > female in fold-change: PDE3A, 9.09 ± 0.57; PDE3B, 3.15 ± 1.91; PDE5A, 2.22 ± 0.1; *n* = 3-7, *p* < 0.05, Figures 1Biii). Two genes, PDE1B and PDE3A, were expressed at higher levels in juvenile males compared with juvenile females (male > female in fold-change: PDE1B, 1.67 ± 0.11; PDE3A, 25.00 ± 0.22; *n* = 4–6, *p* < 0.05, Figures 1Biv).

### Reproductive Maturity-specific Differences in PDE1-5 mRNA Expression

To determine differences of PDE mRNA levels between adults and juveniles, the fold-change of gene expression was calculated using 2^−ΔΔCt^ where ΔΔCt = ΔCt_adult_—ΔCt_juvenile_ when the target gene levels for adults were greater compared with juveniles and vice versa.

In arterioles, expression of four isoenzymes, PDE1C, PDE3A, PDE3B, and PDE4A, was greater in adult males compared with juvenile males (adult male > juvenile male, fold-change: PDE1C, 7.00 ± 0.11; PDE3A, 14.29 ± 0.02; PDE3B, 7.91 ± 0.32; PDE4A, 2.63 ± 0.04; *n* = 4–6, *p* < 0.05, Figures 1Ci). In females, PDE3A expression was higher in adult females compared with juvenile females (adult female > juvenile female, PDE3A, 6.18 ± 0.29; *n* = 7, *p* < 0.05). Expression levels of PDE1A and PDE1C were greater in juvenile females than in adult females (juvenile female > adult female, fold-change: PDE1A, 1.84 ± 0.34; PDE1C, 2.00 ± 0.29; *n* = 4–6, *p* < 0.05, Figures 1Cii).

In venules, there was only one gene, PDE4D, where expression was higher in adult males relative to juvenile males (adult male > juvenile male PDE4D, 1.96 ± 0.11-fold; *n* = 6, *p* < 0.05, Figures 1Ciii). Expression levels of PDE1B, PDE4A, and PDE4D were higher in adult relative to juvenile females (adult female > juvenile female, fold-change: PDE1B, 1.92 ± 0.15; PDE4A, 2.18 ± 0.08; PDE4D, 1.96 ± 0.11; *n* = 5-6, *p* < 0.05, Figures 1Civ).

### PDE Protein Expression

**FIGURE 2:**
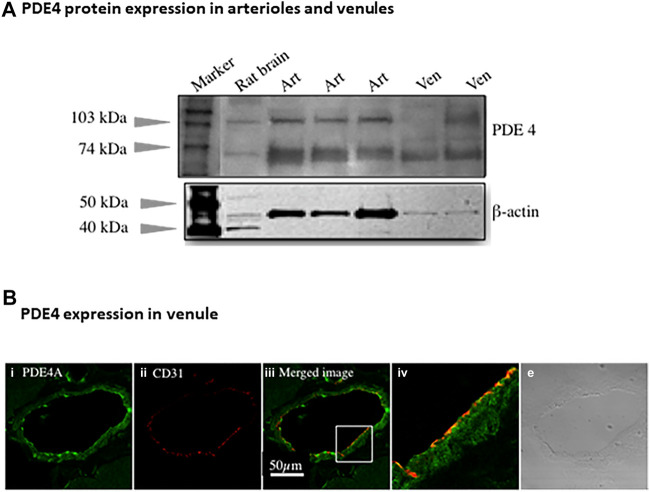
PDE4 protein expression in skeletal muscles arterioles and venules. **(A)** The representative immunoblotting using non-selective PDE4 antibody shows PDE4 protein expressed in arterioles and venules isolated from rat abdominal skeletal muscles. Rat brain was used as a positive control. The expression of β-actin protein was used as a loading control for corresponding vessels. Art: arteriole; Ven: venule. **(B)** The representative immunofluorescence imaging was acquired using scanning confocal microscopy. The sectioned rat abdominal skeletal muscle was stained with PDE4A primary and Alexa-488 (green color) secondary antibodies **(i)** as well as CD31 (an endothelial marker) primary and Alexa-568 (red color) secondary antibody **(ii)**. The images of **(i)** and **(ii)** were superimposed **(iii)**, in which the yellow color indicates co-localization of PDE4A and CD31. The section in the small box of image c was enlarged **(iv)**, showing the co-localization of PDE4A and CD31 molecules. The transmitted light image **(v)** shows the venule of skeletal muscle tissue. The scale bar represents 50 µm for all the images except the image **(iv)**.

Immunoblotting assay using antibody against pan-PDE4 family verified the presence of PDE4 protein in arterioles and venules isolated from rat abdominal skeletal muscles (Figure 2A). There were two major bands indicated by arrows due to different isoforms of PDE4 identified by pan-PDE4 antibody. We used immunofluorescence staining and confocal laser scanning microscopy analysis (Figure 2B) to determine the distribution of PDE4A protein in rat abdominal skeletal muscle venules. The green color in the image shown in Figure 2B represents the presence of PDE4A and the red color represents CD31 (PECAM-1) protein used as an endothelial cell marker. As two images are superimposed, the yellow color indicates the relative co-localization of PDE4A and CD31, consistent with the presence of PDE4A in the venule endothelium.

## Discussion

The study is the first to demonstrates mRNA expression of five PDE (1–5) families in skeletal muscle microvessels (arterioles and venules) in adult and juvenile rats of both sexes. Contrary to our initial hypothesis both age and sex influenced the expression levels of the PDEs central to cardiovascular function. Expression levels of all 10 isoenzymes of PDE1-5 can be divided into three categories: 1) high level: PDE5A; 2) modest level: PDE1A, PDE1C, PDE2A, PDE4B and PDE4D; 3) low level: PDE1B, PDE3A, PDE3B, and PDE4A. With respect to sex, analysis of sexual dimorphism in PDE1-5 expression reveals that 1) in adults, expression levels of five genes (PDE 1C, 3A, 3B, 4B, and 5A) in arterioles and three genes (PDE3A, 3B, and 5A) in venules are greater in males when compared with females (Figures 1Bi,iii); 2) in juveniles, while two genes (PDE1B and 3A) are higher in males compared with females in venules (Figures 1Biv), only PDE1A is greater in females compared with males in arterioles (Figures 1Bii). With respect to age, reproductive maturity-related differences in expression level of isoenzyme were found: 1) in males, four isoenzymes (PDE1C, 3A, 3B, and 4A) in arterioles (Figures 1Ci) and one gene (PDE4D) in venules (Figures 1Ciii) are greater in adults than juveniles; 2) in females, one gene (PDE3A) in arterioles (Figures 1Cii) and three genes (PDE1B, 4A, and 4D) in venules are greater in adults than juveniles while two genes (PDE1A and 1C) are greater in juveniles compared with adults (Figures 1Civ).

An overall implication from the comparisons between sex- and age-is that increases in testosterone with sexual maturity mediates the higher expression levels of arteriolar PDE1C, 3A, and 3B in adult males than in adult females or juveniles of either sex. It is worth noting the complexity of teasing out the contribution of gonadal hormones to sex differences. In the simplest paradigm, sexual dimorphisms between juvenile males and females stems from two components: 1) increased gonadal hormone levels during embryonic gonadal organ differentiation, although following the first days of life, circulating gonadal hormone levels are low and comparable between juvenile males and females; 2) sex chromosome complements (XY or XX) (Arnold and Burgoyne, 2004; Grimm et al., 2021). Obviously, the roles for sex steroid production and its change with age, and the contribution of the myriad of sex hormone isoforms are beyond the scope of the present work and require much closer inspection.

*PDE1.* This study demonstrates the robust PDE1A and 1C expression relative to PDE1B in skeletal muscle microvessels, supporting functional studies on vasomotion for PDE1A and proliferation for PDE1C (Chan and Yan, 2011). Increased PDE1A levels in the small mesenteric artery mediates angiotensin-induced hypertension (Giachini et al., 2011). PDE1A had also been identified as responsible for pulmonary hypertension in pulmonary arterioles (Schermuly et al., 2007). PDE1 inhibitor-induced decrease in total pulmonary resistance in pulmonary hypertensive rats is mediated by reduction of cGMP due to stimulation of cGMP hydrolysis. Moreover, the upregulation of PDE1A has been shown to be associated with nitrate tolerance that occurs during extended treatment using nitroglycerin as a vasodilator (Kim et al., 2001). PDE1C has been demonstrated to contribute to proliferation and migration of vascular smooth muscle cells associated with neointimal hyperplasia (Cai et al., 2015) as well as pulmonary hypertension (Schermuly et al., 2007). Unlike PDE1A and 1C, PDE1B expression in skeletal muscle arterioles is relatively low, consistent with detection of PDE1B in microvasculature of rat skeletal muscle via immunofluorescence assay, but not by highly sensitive RT-PCR method using whole skeletal muscle (Genders et al., 2011). The RT-PCR assay is supposed to be able to detect rare transcripts and small changes in gene expression (Pfaffl, 2001).

Notably, this study demonstrates a higher venular PDE1A expression in juvenile females relative to both juvenile males and adult females (Figures 1Bii and 1C-b). This outcome is at odds with our previous work, using primary rat skeletal muscle microvascular endothelial cells, where PDE1A expression was found to be higher in males than females (Wang et al., 2010). The discrepancy most likely reflects the presence of abundant smooth muscle cells in intact arterioles. The smooth muscle protein levels and functional impacts on arteriolar vasomotion and proliferation remain to be investigated. Further, it remains to be determined how endothelial and vascular smooth muscle compartmentalization of the cyclic nucleotides is achieved and how it influences function.

*PDE2A.* The findings demonstrate comparable PDE2A expression for skeletal muscle arteriole and venules, respectively, regardless of sex and reproductive maturity of rats. Those findings are consistent with our previous study which shows equivalent levels in microvascular endothelial cells between males and females as well as protein expression in skeletal muscle arterioles revealed by immunoblotting and immunofluorescence staining (Wang et al., 2010). Those data are also supported by results derived from human microvessels, but differ from the findings in large arteries such as the aorta and pulmonary arteries (Sadhu et al., 1999).

Functional studies have revealed involvement of PDE2A in endothelial barrier dysfunction and hyperpermeability induced by TNFα (Seybold et al., 2005) and higher doses of a nitric oxide donor or thrombin (Surapisitchat and Beavo, 2007). PDE2A hydrolyzes both cAMP and cGMP. At physiological concentrations, cGMP can regulate PDE2A-meidated cAMP activity, and stimulated PDE2A-mediated cAMP hydrolysis up to 50-fold (Stenson et al., 2010), which may be responsible for hyperpermeability by stimulation of TNFα, NO donors, or thrombin.

*PDE3.* Consistent with the findings in microvascular endothelial cells (Netherton and Maurice, 2005; Wang et al., 2010), this study found PDE3 expression in intact arterioles and venules of skeletal muscles under basal conditions. In comparison to PDE1, 2A, 4, and 5A family, PDE3 expression levels are relatively low, evidenced by both our previous and this study. Despite low expression levels, functional data have demonstrated that PDE3 affects profoundly vascular tone, angiogenesis (Sanada et al., 2016), and vascular barrier integrity (Surapisitchat et al., 2007; Francis et al., 2011; Surapisitchat et al., 2011). Inhibitors of PDE3 are used as pharmacological therapeutic treatment for intermittent claudication associated with peripheral arterial occlusive disease (Francis et al., 2011). Little is known, however, of sex-specific differences in PDE expression in microvessels and vascular cells.

We found that in adults, the expression levels of PDE3A and 3B are greater in adult males compared with adult females in both arterioles and venules (Figures 1Bi and Figures 1Biii), indicating a sex-specific difference. Our previous study using microvascular endothelial cells showed greater expression of PDE3B in cells derived from females relative to males (Wang et al., 2010). Again, the disparity in PDE3B expression between cultured endothelial cells and intact arterioles may be partially attributed to multiple vascular cell types constituting arterioles, including endothelial cells, smooth muscle cells, and other connective tissue cells.

Using TaqMan RT-PCR, this study still showed large standard errors for PDE3 gene expression in microvessels. This is possibly caused by relatively low expression of PDE3A and PDE3B and small changes in the expression levels among biological individuals may be amplified dramatically. Since PDE3 is involved in the regulatory mechanisms of a myriad of vascular functions, from regulation of vascular tone to maintenance of barrier function, studying not only the expression level in cells or tissue but also its microdomain and activity duration in cells would better characterize PDE3.

*PDE4.* In this study we found robust PDE4 expression in intact arterioles relative to PDE2 and PDE3 which are involved in regulating cAMP hydrolysis, consistent with our previous data for microvascular endothelial cells (Wang et al., 2010) and results from Maurice’s group (Netherton and Maurice, 2005). Substantial *in vivo* evidence supports that PDE4 promotes pro-inflammatory mediator-induced endothelial barrier dysfunction by reducing cAMP level through promoting its hydrolysis (Rampersad et al., 2010; Lin et al., 2011; Schick et al., 2012). In addition, PDE4 has been shown to critically determine the localization, magnitude, and duration of subcellular cAMP compartmentalization (Lynch et al., 2005; Creighton et al., 2008; Stangherlin and Zaccolo, 2012). Among three isoenzymes of PDE4 (A, B, D), PDE4B and PDE4D mRNA are abundant in arterioles and venules regardless of sex and age of rats (Figure 1A). In complementary experiments, the presence of PDE4 protein in arterioles and venules isolated from skeletal muscles was validated by immunoblotting assay (Figure 2A, using antibody against pan-PDE4). Immunofluorescence assay revealed that venular endothelial cells express PDE4 (Figure 2B), consistent with results using primary cultured rat microvascular endothelial cells (using antibody against PDE4D) (Wang et al., 2010).

Sexual dimorphism in PDE4 has been reported. In this study, we found greater PDE4B expression in adult males than adult females in arterioles (Figure 1B), that was not recapitulated in juveniles or venules of either age group. In rat cardiac ventricular tissue, greater expression of PDE4B transcripts is found in females relative to males (Parks et al., 2014). In humans, it has been shown that the correlation between PDE4D gene expression and carotid atherosclerosis (assessed by intima-media thickness and plaque index) exists only for men, not women (Liao et al., 2010). Of interest it is three isoenzyme transcripts (PDE4A, B, and D) that are greater in adult relative to juvenile male rats (Figure 1C). Herein, we reported age-dependent expression of PDE4D in venules for both males and females (Figures 1Ciii,iv, adults > juvenile).

*PDE5A* **.** The PDE5A expression in both arterioles and venules was robust and far greater than other isoenzyme levels (Figure 1A), implicating the important roles of PDE5A in the regulation of microvascular functions. Upregulation of PDE5A expression in smooth muscle cells induced by angiotensin II decreases cGMP signaling, contributing to vasoconstriction (Kim et al., 2005) while downregulation of PDE5A is associated with human thoracic aortic aneurysms (Cesarini et al., 2019). Inhibition of cGMP-specific PDE5A and subsequently elevated cGMP have been used as the therapeutic strategies for improving tissue perfusion *via* dilation of arterioles in erectile dysfunction, pulmonary hypertension, ischemia/reperfusion injury, myocardial infarction, heart failure, and stroke (Francis et al., 2011). Moreover, PDE5A was demonstrated to promote endothelial proliferation, migration, and capillary-like tube formation during ischemia of the mouse hind limb (Senthilkumar et al., 2007; Sahara et al., 2010; Cesarini et al., 2020).

Given tissue- and vessel-dependent heterogeneous functions of endothelial cells, knowing PDE5A distribution and expression in corresponding tissues/vessels is important for understandings of mechanisms underlying PDE5A vascular function. For instance, PDE5A has been shown in rat pulmonary arterial, but not pulmonary microvascular endothelial cells (Zhu et al., 2005). Consistent with important role of PDE5A, we have shown robust expression of PDE5A in both microvascular endothelial cells (Wang et al., 2010) and intact microvessels of rat abdominal skeletal muscles. Furthermore, sex differences in PDE5A expression are observed in both arterioles and venules isolated from adult rats (male > female, Figure 1B).

**FIGURE 3:**
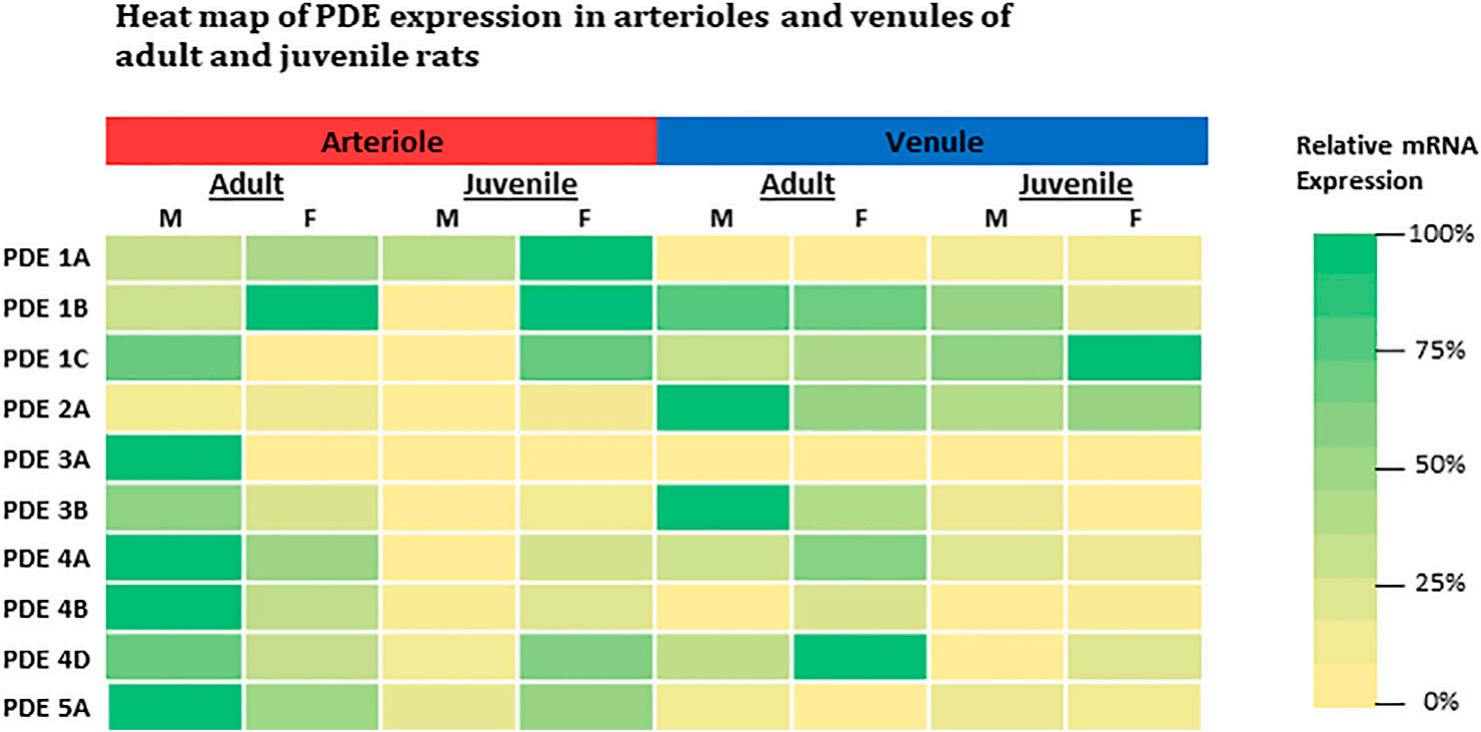
Heat-map comparison for expression levels of PDE1-5 in both arterioles and venules across adult and juvenile male and female rats. For each gene, expression level was compared across all microvessels regardless of its origin (age, sex, and microvessel type) by normalizing each expression level to the highest mRNA level (2^−∆∆Ct^, where ∆∆Ct = ∆Ct_each_–∆Ct_highest_). The color is defined as the highest expression level in green (100%) and the lowest in yellow (0%). M: male; F: female.

In general, our findings demonstrate the basal expression levels of ten PDE1-5 isoenzyme transcripts in intact skeletal muscle arterioles and venules isolated from four different groups of rats. The intrinsic factors of sex and age (reproductive maturity) are associated with expression levels of some PDE1-5, which is partially consistent with our hypothesis. The relative expression of each gene for both arterioles and venules among four groups of rats is summarized in the heat map (Figure 3). Overall, in arterioles, male adults express relatively high levels for seven genes (PDE1C, 3A, 3B, 4A, 4B, 4D, and 5A); in venules, the levels of PDE2A and 3B in adult males and PDE4D in females are the highest across sex, age, and vessel types. Juvenile females express relatively high levels of PDE1 (1A, 1B, and 1C) in arterioles and PDE1C in venules. Therefore, not all results derived from studies using adult males can be extrapolated to adult females or juveniles. Knowing the different baselines of PDE1-5 mRNA expression can provide insights into cAMP- and cGMP-dependent signaling mechanisms of arteriolar and venular functions under physiological and pathological conditions. Also, when PDE levels are changed in disease states it is imperative to know the expression levels under normal conditions as this study most certainly demonstrates the fallacy of assuming no differences in the basal state with sex and/or with age. Further our data point to the importance of assessing whether pharmacological strategies targeting a particular class of PDE are equally effective among family members given the sex- and age-differences in expression noted herein. Finally, studies on PDEs expression in other types of muscles besides the abdominal muscle as well as sex-specific or age-specific differences in molecular activities and vascular functions mediated by PDE1-5 remain to be determined. We understand that activities of cAMP and cGMP can be determined by not only degradation or turnover rate through PDEs but also synthesis rate such as eNOS-NO-cGMP signaling pathway. To the best of our knowledge, cAMP and cGMP synthesis rate also can be affected by the sex and age.

## Perspectives

Cyclic nucleotides as second messengers critically mediate a host of different physiological and pathological aspects of vascular functions. PDEs intimately regulate the level of cyclic nucleotides as they are the sole cytosolic enzymes hydrolyzing cAMP and cGMP. The trivial differences in PDE transcripts between sexes or age (reproductive maturity) states can confound regulatory mechanisms of microvascular functions from vascular tone, to blood flow distribution, amount, and exchange, profoundly impacting the development and progression of multiple diseases associated with vascular inflammation and dysfunction.

## Data Availability

All datasets for this study are included in the article/supplementary material.
